# Pathways of Distinction Analysis: A New Technique for Multi–SNP Analysis of GWAS Data

**DOI:** 10.1371/journal.pgen.1002101

**Published:** 2011-06-09

**Authors:** Rosemary Braun, Kenneth Buetow

**Affiliations:** 1 Laboratory of Population Genetics, National Cancer Institute, National Institutes of Health, Bethesda, Maryland, United States of America; 2 Center for Biomedical Informatics and Information Technology, National Cancer Institute, National Institutes of Health, Bethesda, Maryland, United States of America; University of California San Diego and The Scripps Research Institute, United States of America

## Abstract

Genome-wide association studies (GWAS) have become increasingly common due to advances in technology and have permitted the identification of differences in single nucleotide polymorphism (SNP) alleles that are associated with diseases. However, while typical GWAS analysis techniques treat markers individually, complex diseases (cancers, diabetes, and Alzheimers, amongst others) are unlikely to have a single causative gene. Thus, there is a pressing need for multi–SNP analysis methods that can reveal system-level differences in cases and controls. Here, we present a novel multi–SNP GWAS analysis method called Pathways of Distinction Analysis (PoDA). The method uses GWAS data and known pathway–gene and gene–SNP associations to identify pathways that permit, ideally, the distinction of cases from controls. The technique is based upon the hypothesis that, if a pathway is related to disease risk, cases will appear more similar to other cases than to controls (or vice versa) for the SNPs associated with that pathway. By systematically applying the method to all pathways of potential interest, we can identify those for which the hypothesis holds true, i.e., pathways containing SNPs for which the samples exhibit greater within-class similarity than across classes. Importantly, PoDA improves on existing single–SNP and SNP–set enrichment analyses, in that it does not require the SNPs in a pathway to exhibit independent main effects. This permits PoDA to reveal pathways in which epistatic interactions drive risk. In this paper, we detail the PoDA method and apply it to two GWAS: one of breast cancer and the other of liver cancer. The results obtained strongly suggest that there exist pathway-wide genomic differences that contribute to disease susceptibility. PoDA thus provides an analytical tool that is complementary to existing techniques and has the power to enrich our understanding of disease genomics at the systems-level.

## Author Summary

We present a novel method for multi–SNP analysis of genome-wide association studies. The method is motivated by the intuition that, if a set of SNPs is associated with disease, cases and controls will exhibit more within-group similarity than across-group similarity for the SNPs in the set of interest. Our method, Pathways of Distinction Analysis (PoDA), uses GWAS data and known pathway–gene and gene–SNP associations to identify pathways that permit the distinction of cases from controls. By systematically applying the method to all pathways of potential interest, we can identify pathways containing SNPs for which the cases and controls are distinguished and infer those pathways' role in disease. We detail the PoDA method and describe its results in breast and liver cancer GWAS data, demonstrating its utility as a method for systems-level analysis of GWAS data.

## Introduction

Genome-wide association studies (GWAS) have become a powerful and increasingly affordable tool to study the genetic variants associated with disease. Modern GWAS yield information on millions of single nucleotide polymorphism (SNPs) loci distributed across the human genome, and have already yielded insights into the genetic basis of complex diseases [1], [2], including diabetes, inflammatory bowel disease, and several cancers [3]–[7]; a complete list of published GWAS can be found at the National Cancer Institute–National Human Genome Research Institute (NCI-NHGRI) catalog of published genome-wide association studies [8].

Typically, the data produced in GWAS are analyzed by considering each SNP independently, testing the alleles at each locus for association with case status; significant association is indicative of a nearby genetic variation which may play a role in disease susceptibility. Genomic regions of interest may also be subject to haplotype analysis, in which a handful of alleles transmitted together on the same chromosome are tested for association with disease; in this case, the loci which are jointly considered are located within a small genomic region, often confined to the neighborhood of a single gene.

Recently, however, there has been increasing interest in multilocus, systems-based analyses. This interest is motivated by a variety of factors. First, few loci identified in GWAS have large effect sizes (the problem of “missing heritability”) and it is likely that the common–disease, common–variant hypothesis [9], [10] does not hold in the case of complex diseases. Second, single marker associations identified in GWAS often fail to replicate. This phenomenon has been attributed to underlying epistasis [11], and a similar problem in gene expression profiling has been mitigated through the use of gene-set statistics. Most importantly, it is now well understood that because biological systems are driven by complex biomolecular interactions, multi-gene effects will play an important role in mapping genotypes to phenotypes; recent reviews by Moore and coworkers describe this issue well [10], [12]. Additionally, the finding that epistasis and pleiotropy appear to be inherent properties of biomolecular networks [13] rather than isolated occurences motivates the need for systems-level understanding of human genetics.

The impact that biological interaction networks have on our ability to identify genomic causes of complex disease is readily apparent. Consider a biologically crucial mechanism with several potential points of failure, such that an alteration to any will confer disease risk. Because no single alteration is predominant amongst cases, none attain a significant association; indeed, it has long been observed that even in histologically identical tumors, only a fraction may share the same set of mutations (see references in [14] for examples). Additionally, in a robust system, multiple alterations may be necessary to alter the activity of an interaction network; here, healthy individuals may share a subset of the deleterious alleles found in cases, and again these loci will not be detected. This complexity, noted by [10], [12]–[14] and others, has generated considerable interest in multi-locus analysis techniques that take advantage of known interaction information.

Several multi-SNP GWAS analysis approaches have been described in the literature. Thorough reviews are provided in [15], [16], and we briefly describe several here. Building on the well-established Gene Set Enrichment Analysis [17] method initially developed for gene expression data, two articles have proposed an extension of GSEA for SNP data [18], [19]. In these techniques, each SNP is assigned a statistic based on a 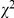 test of association with the phenotype; a running sum is then used to assess whether large statistics occur more frequently amongst a SNP set of interest than could be expected by chance. While GSEA-type approaches have proven quite useful, their reliance on single-marker statistics means that systematic yet subtle changes in a gene set will be missed if the individual genes do not have a strong marginal association. In the case of a purely epistatic interaction between two SNPs in a set, the set may fail to reach significance altogether.

To address this issue, Yang and colleagues proposed SNPHarvester [20], designed to detect multi-SNP associations even when the marginal effects are weak. To reduce the search space of possible multi-SNP effects, SNPHarvester [20] first removes any SNPs with univarite significance. Using a novel searching algorithm, they identify groups of 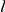 SNPs that show association with status in a 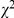 test with 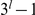 degrees of freedom. While this approach can reveal epistatic effects that would be missed by the GSEA-type schemes [18], [19], it has other drawbacks. First, the combinatorial explosion of SNP groups puts a limit on the number of SNPs 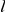 that may simultaneously be examined. Second, the the arbitrary groupings of SNPs and the exclusion of SNPs with marginal effects can make the biological interpretation of the analysis results difficult.

The notion that cases will more closely resemble other cases than they will controls has motivated a number of interesting distance-based approaches for detecting epistasis. Multi-dimensionality reduction (MDR) has been proposed and applied to SNP data [21]–[23]. In this technique, sets of 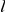 SNPs are exhaustively searched for combinations that will best partition the samples by examining the 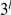 cells in that space (corresponding to homozygous minor, heterozygous, or homozygous major alleles for each locus) for overrepresentation of cases. While this method both finds epistatic interactions without requiring marginal effects and can be structured to incorporate expert knowledge, it is limited by the fact the the total number of loci to be combinatorially explored must be restricted to limit computational cost. To address this, an “interleaving” approach in which models are constructed hierarchically has been suggested [22] to reduce the combinatorial search space. A recent and powerful MDR implementation [24] taking advantange of the CUDA parallel computing architecture for graphics processors has made feasible a genome-wide analysis of pairwise SNP interactions. Still, MDR remains computationally challenging, such that expanding the search to other SNP set sizes (rather than restricting to pairwise interactions) can be impeded by combinatorial complexity if an exhaustive search is to be performed.

In order to narrow down the combinatorial complexity of discovering SNP sets using techniques such as MDR, feature selection may be employed. Of particular importance here is the distance-based approach of the Relief family of algorithms [25]–[28]. These are designed to identify features of interest by weighting each feature through a nearest-neighbor approach. The weights are constructed in the following way: for each attribute, one selects samples at random and asks whether the nearest neighbor (across all attributes) from the same class and the nearest neighbor from the other class have the same or different values from the randomly chosen sample. Attributes for which in-class nearest neighbors tend to have the same value are weighted more strongly. Because the distances are computed across all attributes, Relief-type algorithms can identify SNPs that form part of an epistatic group and they provide a means of filtration that does not have the drawbacks of other significance filters.

While these methods have so far been applied to finding small groups of interacting SNPs, one may instead be interested in whether cases and controls exhibit differential distance when considering a large number of genes. A multi-SNP statistic has been proposed in the literature [29]–[31] for determining whether an individual of interest is on average (across a large number of SNPs) “closer” to one population sample than to another. The method, originally proposed by Homer [29], is motivated by the idea that a subtle but systematic variation across a large number of SNPs can produce a discernible difference in the closeness of an individual to one population sample relative to another. While this statistic was originally designed to identify the proband as a member of one of the population samples, it was shown in [30] that out-of-pool cases from a case-control breast cancer study were in general closer (as defined by the statistic presented in [29]) to in-pool cases than they were to in-pool controls, suggesting that the combination of multiple alleles has the potential to distinguish cases from controls.

Building on these ideas, we present a new technique that finds pathway-based SNP-sets that differentiate cases from controls; we call this method Pathways of Distinction Analysis (PoDA). In PoDA, SNP sets are defined based on known relationships (e.g., SNPs in genes sharing a common pathway), and thus incorporate expert knowledge to reduce the search space and provide biological interpretability. Motivated by the differential “closeness” of cases and controls as discussed about and as observed in [30], we hypothesize that if the SNPs come from a pathway that plays a role in disease, there will be greater in-class similarity than across-class similarity in the genotypes for those SNPs; i.e., a case will show greater genetic similarity to other cases than to controls for the SNPs on a disease-related pathway, but will be equidistant for the SNPs on a non-disease-related pathway. Based on this notion, PoDA seeks to identify pathways for which differential heterogeneity is exhibited in cases and controls. In each pathway, PoDA returns a statistic 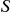 for each sample that quantifies that sample's distance to the remaining cases relative to its distance to the remaining controls for a given pathway's SNPs. PoDA then examines whether the distributions of 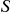 for the controls differ from those of the cases by computing and testing for significance a Pathway Distinction Score 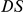 that quantifies the differences in case and control 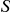 distributions. In this manuscript, we detail the PoDA method and report the results of its application to two data sets.

As we will describe, PoDA improves and complements existing approaches in a number of respects. First, it permits the investigation of arbitrarily large pathways, circumventing the dimensionality issues that are encountered with MDR and SNP-Harvester. Second, it is able to detect pathways that contain an over-abundance of highly-significant markers as well as pathways whose markers have a small but consistent association that would be missed by GSEA-type approaches. Finally, it uses a leave-one-out technique to return for each sample an unsupervised relative distance statistic that can then be used to model disease risk via logistic regression. In addition to providing an effect size for the pathway, this allows the odds of disease for new samples to be obtained by computing its relative distance statistic with respect to the known samples and applying the model.

## Methods

Following our conjecture that SNPs associated with the genes in a pathway involved in disease will exhibit more within-group similarity than across-group similarity, we propose Pathways of Distinction Analysis (PoDA), a method designed to address the following questions:

- Given some set of SNPs, do we find that, on average, cases are “closer” to other cases than to controls (or that controls are “closer” to other controls than to cases)?
- If we look for these distinctions systematically over all SNP-sets of potential interest, can we use it to single out SNP-sets which may be associated with disease?

In PoDA, a set of SNPs are selected, and for each sample we compute whether it is closer to the pool of remaining cases or controls across that SNP set, using the relative distance statistic described below. Once this is done for every sample, the distribution of the relative distance statistic is compared in the cases and controls using a nonparametric statistic, addressing the first question above. This may be carried out amongst all SNP sets of interest, adjusting the 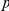-value for the multiple hypotheses, to find SNP sets for which cases more strongly resemble the population of remaining cases while controls more strongly resemble the population of remaining controls.

We begin with a discussion of how we measure the relative distance of an individual to the other cases vs. other controls. A simple but computationally intensive approach is to represent each sample by a vector in an 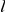-dimensional space, where 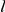 is the number of SNPs in the group of interest. One can then compute, between each sample pair, their distance in this 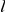-dimensional space using a Euclidean, Manhattan, or Hamming metric. For each sample, we would define its relative distance statistic as the mean of its distance to other controls minus the mean of its distance to other cases; a sample that is more similar to cases will exhibit a positive statistic, whereas one that is more similar to other controls will exhibit a negative statistic. For the given SNP set, we would then have for each sample a value quantifying its relative distance that was computed without knowledge of that sample's class (i.e., using a leave-one-out scheme) and could then be used in further tests. By doing this for all pathways of interest, one derives a relative distance value for each sample in each pathway.

This brute-force approach, while conceptually clear, has two significant drawbacks. The first is that the distance computation is 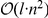 where 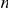 is the total number of samples in the study–a considerable undertaking, particularly if many SNP sets are to be analyzed, and one that becomes exceedingly troublesome in the context of permutation tests. The second drawback is that because we are taking the mean of the distances, a sample that is situated squarely within a cluster of cases may have a large case-distance value due to the dispersion of cases around it. Both of these issues are circumvented by instead considering the relative distance to the *centroids* of the cases and controls in the 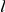-dimensional space, a computation that can be performed in 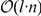 for all 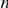 samples. It is this approach that PoDA employs, as follows:

In [29], [30], the authors consider a measure of individual 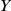
's distance to two population samples 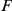 and 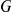 at locus 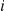,
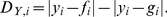
(1)where 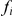 and 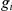 are the minor allele frequencies (MAFs) of SNP 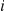 in samples 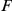 and 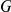, and 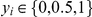 is 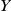
's genotype at 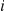 corresponding to homozygous major, heterozygous, and homozygous minor alleles, respectively (i.e., the frequency of minor allele in that individual. The first term quantifies how different 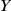
's MAF is from 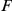
's for a given locus 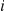; the second term quantifies how different 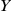
's MAF is from 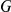
's at locus 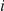; and so 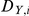 gives the distance of 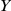 relative to 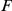 and 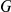 at locus 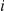. Since the minor allele frequencies 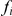 and 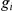 are computed by averaging the genotypes (again, written as 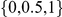) in samples 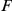 and 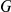 respectively, it is clear that 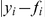 is the distance from 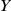 to the centroid of 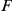 along the coordinate 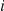 (and likewise for the 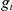 term). It can be seen from Eq. 1 that positive 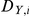 implies that 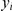 is closer to 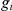 than to 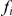, and that negative 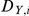 implies that 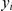 is closer to 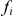 than to 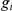.

By computing 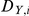 at each locus 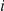 and taking the standardized mean across the 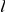 loci, [29] obtain a distance score 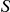 which quantifies how close 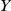 is relative to 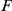 and 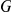 across all 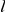 loci under consideration,
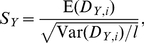
(2)where 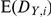 denotes the mean of 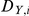 across all loci 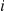. That is, 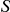 provides a means to quantify whether 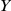
's MAFs are closer to 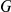
's MAFs or 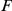
's MAFs on average for the loci under consideration. It is instructive to consider the geometrical interpretation of Eq. 2. Is clear upon inspection that the numerator in Eq. 2 is equal, up to a factor of 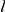, to the difference in Manhattan distances between 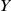 and the (nonstandardized) 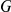 centroid and 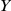 and the (nonstandardized) 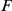 centroid; in this sense, Eq. 2 resembles a nearest-centroid classifier. However, the denominator scales the relative distances by their variance across the 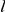 SNPs; that is, a sample 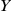 who is consistently closer to 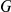 than to 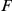 for each of the 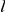 SNPs will obtain a higher 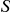 than an individual who is variously closer to either across the 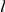 SNPs under consideration.

By assigning the (non-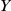) controls as 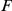 and the (non-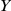) cases as 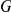, we can compute a statistic 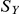 quantifying 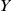
's distance to other cases relative to 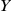
's distance to other controls. If we then apply this systematically to all individuals in the study population (removing that individual, computing the MAF's 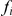 and 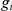 for the remaining individuals who comprise 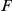 and 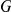, and then computing 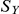 in a leave-one-out manner), we can obtain distributions of 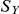 statistics in cases and controls that may be compared. Here, the null hypothesis is that case and control 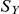 distributions do not differ, with the alternative hypothesis that the cases have higher 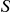 values than do controls, i.e., that they are closer (via Eqs. 1–2) to other cases than are controls.

We can use 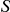 in the following manner to answer the questions posed above by applying it in a leave-one-out manner in each pathway:

1. For a given pathway 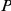, select the 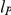 SNPs associated with that pathway;
2. For every sample 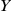, remove 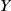 from the case or control group as needed, and compute 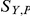 with respect to the remaining cases and controls using the SNPs chosen in step 1.
3. Quantify the differences in distribution of 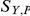
's for the case samples versus that of the controls and test for significance.

By systematically carrying out the above steps on all pathways of interest, we can identify pathways for which there appears to be differential homogeneity in cases and controls, indicating that the pathway may play a disease-related role. The details of the algorithm are explained below, and summarized in Table 1.

**Table 1:** Procedure for Pathways of Distinction Analysis.

1.	For a each pathway 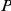 , select all associated genes from pathway database such as PID [32];
2.	For each gene on the pathway, select associated SNPs (e.g., using dbSNP) and choose the one with the strongest association with case status, determined using Fisher's exact test;
3.	For each sample 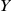 in the GWAS, select the controls 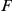 and cases 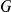 which do not include it, compute MAFs 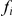 and 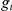 for the SNPs 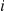 selected in step 2, and compute 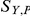 for each sample 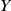 ;
4.	Compare the distribution of 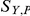 obtained in step 2 for cases to that of controls by computing the Wilcoxon statistic 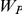 based on the 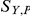 for that pathway;
5.	Repeat steps 2–5 using permuted case/control labels, and normalize 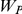 by the distribution of 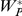 obtained with permuted labels, yielding the distinction score 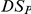 ;
6.	Compare the distinction score 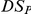 obtained in step 5 to that obtained for random sets of 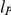 genes, where 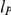 is the number of genes in the pathway of interest.

In [30], we examined Eqs. 1–2 and found that the magnitude of 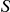 is influenced both by the MAF differences 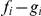 (that is, how distant the centroids of 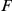 and 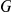 are) and by correlations between the SNPs under consideration (due to the penalization for variance in 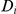 provided by the denominator of Eq. 2. These properties are extremely well-suited to the application we propose: pathways with few highly-significant SNPs will yield large 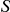 differences (due to the influence of 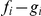), as will pathways with SNPs that are highly correlated yet have subtle individual MAF differences, corresponding to the concerted action of multiple SNPs.

At the same time, however, we wish to ensure that the pathways we select as having differential 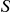 are not being influenced large LD blocks covered by the SNPs in the genes on the pathway. That is, we wish to ensure that the SNP correlations which drive 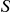 are reflective of epistatic effects between different genes rather than recombination events within a gene. To this end, we select a single SNP to represent each gene, based on the desire to detect multi-*gene* rather than multi-SNP effects.

In practice, SNPs are selected as follows: for each pathway represented in the Pathway Interaction Database [32] (PID, http://www.pid.nci.gov, containing annotations from BioCarta, Reactome, and the NCI/Nature database [32]) and KEGG [33], we select the associated genes. Using dbSNP [34], we retrieve the SNPs associated with the pathway genes that are present in the data, excluding those with 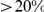 missing data or with minor allele frequency 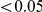 in either case of control group. We necessarily exclude pathways for which only one gene is probed by the remaining SNPs. Because we are interested in 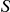 values that are driven by correlations *across* genes (and not in individual genes covered by many SNPs with high LD), we select for each gene its most significant SNP in a univariate test of association (Fisher's exact test). It should be noted here that while the SNP chosen for each gene is the most significant of that gene's SNPs, it is *not* necessarily significantly associated with disease. Our goal here is not to filter based on SNP significance, but rather to select, for each gene, a single marker that is as informative as possible.

Having selected the SNPs of interest, we compute 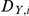 at each locus for every sample by selectively removing it and comparing it to the remaining cases and controls, as described above. For each pathway 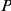, we compute 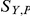 for 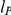 the SNPs 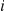 that comprise it, yielding a distribution of 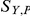 for cases and another distribution for controls. The difference in the location of the case and control 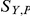 distributions is then quantified nonparametrically by computing the Wilcoxon rank sum statistic, defined as
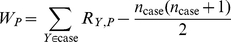
(3)where 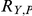 is the rank of 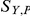 amongst all samples 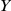 for a given pathway 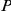. Eq. 3 thus quantifies, non-parametrically, the degree to which cases are “closer” to other cases and controls “closer” to other controls across a set of SNPs for all individuals in the GWAS.

**Figure 1:**
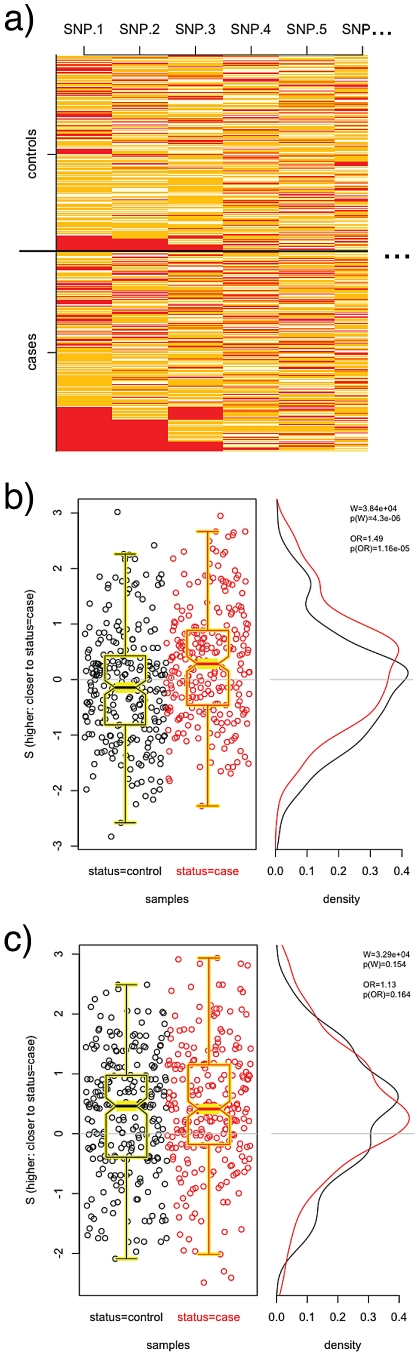
PoDA applied to simulated data. Alleles at 50 loci for 250 cases and 250 controls were simulated such that each SNP was in HWE and not associated with case status, but homozygous minor (red) at both loci 1 and 2 or 1 and 3 yielded a three-fold relative risk (a). A 12-SNP pathway comprising SNPs 1–12 shows differential 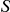 distributions (b); a random 12-SNP pathway does not (c). Boxplots are overlayed on the scatterplots of 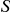 for clarity.

To illustrate the above, we consider a simulated GWAS of 250 cases and 250 controls and 50 SNPs, shown in Figure 1, and ask whether we are able to detect a 12-SNP pathway in which a subset of SNPs appear to have an epistatic interaction. Alleles were simulated as binomial samples from a source population with MAFs ranging from 0.1 to 0.4 across the 50 SNPs, and case labels were assigned such that a combintion of homozygous minor alleles at SNPs 1 and 2 or 3 (i.e., 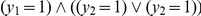) conferred a three-fold relative risk, mimicking an epistatic interaction between SNPs 1 and 2 and SNPs 1 and 3 (Figure 1a). Alone, none of the 50 SNPs showed any association with case status, nor was any SNP significantly out of HWE in either cases or controls. However, the “shared pattern” in SNPs 1–3 is such that a 12 SNP pathway comprising SNPs 1–12 yields greater 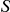 in cases than in controls as can been seen in Figure 1b, while a random 12 SNP pathway selected from the 50 SNPs (that includes SNP 3, but neither SNP 1 or 2) shows no difference in 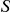 values as seen in Figure 1c.

While the Wilcoxon statistic 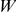 is normal in the large-sample limit and can be directly compared to a Gaussian, to truly evaluate the significance of 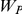 for a given pathway 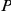, we must address two sources of bias: the number of SNPs per gene, and the size of the pathway. To address these issues, we introduce a normalized Pathway Distinction Score 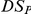 that we test for significance using a resampling procedure.

First, we expect that because we have selected for each gene the single most informative SNP, we are pre-disposed to seeing higher 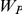 for pathways that contain large genes. Because large genes will be more likely to contain highly-significant SNPs by chance, the concern has been raised that [18], [35] selecting the single most significant SNP as a proxy for the gene (as is done here) will lead to a bias toward pathways that contain an abundance of large genes. To account for this, we follow the approach in [18] and normalize the score via a permutation-based procedure. First, we permute the phenotype labels and in each permutation recalculate 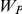 as described above, but using the permuted case and control labels. The permuted labels are used both to select the most informative SNP per gene and to compute 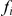, 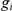, and 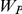 in Eqns. 1–3). This yields a distribution of 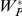 under the null hypothesis that the magnitude of 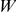 is independent of the true case/control classifications. We then normalize the true 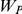 by the distribution from the permutation procedure, yielding a Distinction Score 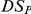 for pathway 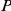 that effectively adjusts for different sizes of genes and preserves correlations of SNPs in the same gene:
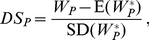
(4)where 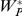 are the set of 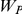 obtained for pathway 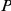 across the permutations. (In practice, 100 permutations are used.) Because the permuted labels are used in the SNP selection, this normalization adjusts for the bias introduced by the fact that large genes have more opportunity to contain significant SNPs by chance. The Pathway Distinction Score 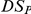 thus provides a model-free, gene-size adjusted metric for quantifying the degree to which cases are “closer” to other cases (higher 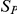) than controls.

To test whether 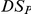 is significant, we note that larger pathways may yield high 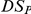 values simply due to the fact that they sample the case anc control differences more thoroughly. Indeed, the question of significance that we wish to address is not simply whether a pathway permits the distinction of cases and controls, but *whether it does so better than a random collection of as many SNPs*, wherein the SNPs are still selected to be the most informative by gene. To account for the fact that the pathways are of differing sizes, significance of the Distinction Score for a given pathway is assessed through resampling by choosing, at random, the same number of SNPs that are present in that pathway (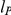) from the total set of most-informative-SNP-per-gene and recomputing 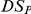 for the random pathway. The 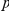 value is obtained by counting the fraction of random 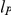-SNP sets which give a larger 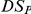 than the true pathway SNPs in 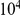 resamplings. In this way, we are able to detect pathways that yield large differences of case and control 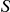 distributions due to their particular SNPs, rather than simply being the result of choosing many SNPs. The 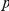 value obtained addresses the question of whether the pathway under consideration permits greater separation of cases and controls than would a random collection of most-informative-SNP-per-gene, i.e., whether there exists a more extreme aggregated effect in that pathway than expected by chance.

## Results

We applied PoDA to 2287 genotypes obtained from the Cancer Genomic Markers of Susceptibility (CGEMS) breast cancer study. The samples were sourced as described in [4]. Briefly, the samples comprised 1145 breast cancer cases and a comparable number (1142) of matched controls from the participants of the Nurses Health Study. All the participants were American women of European descent. The samples were genotyped against the Illumina 550K arrays, which assays over 550,000 SNPs across the genome.

We also applied it to a smaller liver cancer GWAS [36] comprising 386 hepatocellular carcinoma (HCC) patients and 587 healthy controls from a Korean population. Samples were genotyped against Affymetrix SNP6.0 arrays, which provides SNP information at approximately one million loci.

### Breast cancer GWAS results

**Figure 2:**
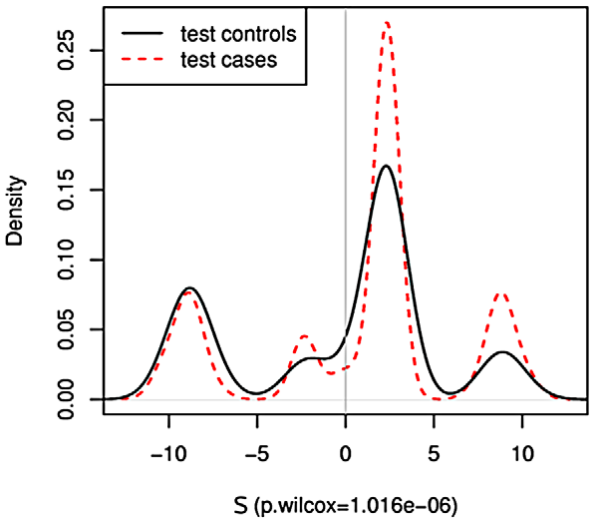
PoDA applied to four highly-significant SNPs. Shown is the distribution of 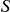 values in CGEMS cases (red) and controls (black) for a SNP-set comprised of four highly-significant SNPs located in the 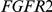 gene [4]. As expected, there is a substantial difference in case and control 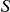 values, with the cases having higher 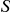 (i.e., closer to other cases) than controls. The discreteness of the distributions are due to the fact that with four SNPs, a finite number of 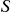 values are possible.

We begin by applying PoDA to the CGEMS breast cancer GWAS data. Having observed (Figure 1) that PoDA performs as expected for the simulated data, we first turn our attention to a simple test in which we select a SNP set comprising the four SNPs in intron 2 of 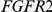 that were reported to show significant association with case status in [4] (rs11200014, rs2981579, rs1219648, rs2420946). We expect to see a strong difference in the test case and test control distributions, and indeed we do: the cases more frequently have positive 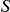 than do controls in Figure 2. (The discrete peaks in the distribution are a result of the fact that with four SNPs there exist fewer available values of 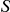.) Using a nonparametric Wilcoxon rank sum test with the alternative hypothesis that cases have greater 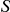 than controls, 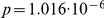 is obtained, confirming our intuition.

We next applied PoDA systematically to the pathways represented in PID [32] using CGEMS data. Associations between genes and SNPs were made using dbSNP build 129 [34]. 1081 pathways were non-trivially covered in the data set; 69453 SNPs in the data could be associated with at least one of the pathways. Because these 69453 SNPs were associated with 4446 unique genes, 4446 were kept in the analysis (the most significant SNP for each gene of interest). The 1081 pathways ranged from 2 to 229 genes, with a mean of 19. 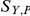 was computed in each pathway 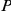 for each of the 2287 samples 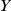 via Eq. 2, and the distinction score 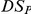 (Eq. 4) quantifying differential 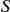 distributions in cases and controls was computed for each pathway. Significance was assessed as described above, by resampling “dummy” pathways of the same length and computing the fraction of greater 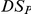 scores.

Because PoDA provides for each sample a measure 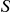 (Eq. 2 of that sample's relative distance from the remaining ones that is obtained without regard to that sample's true class membership, we can use the 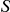 value as a metric by which to predict the odds of disease. Here, we construct a logistic regression model of case status as a function of 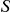 to obtain the odds ratio. 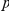-values were adjusted for the multiplicity of pathways using FDR adjustment [37], [38].

**Figure 3:**
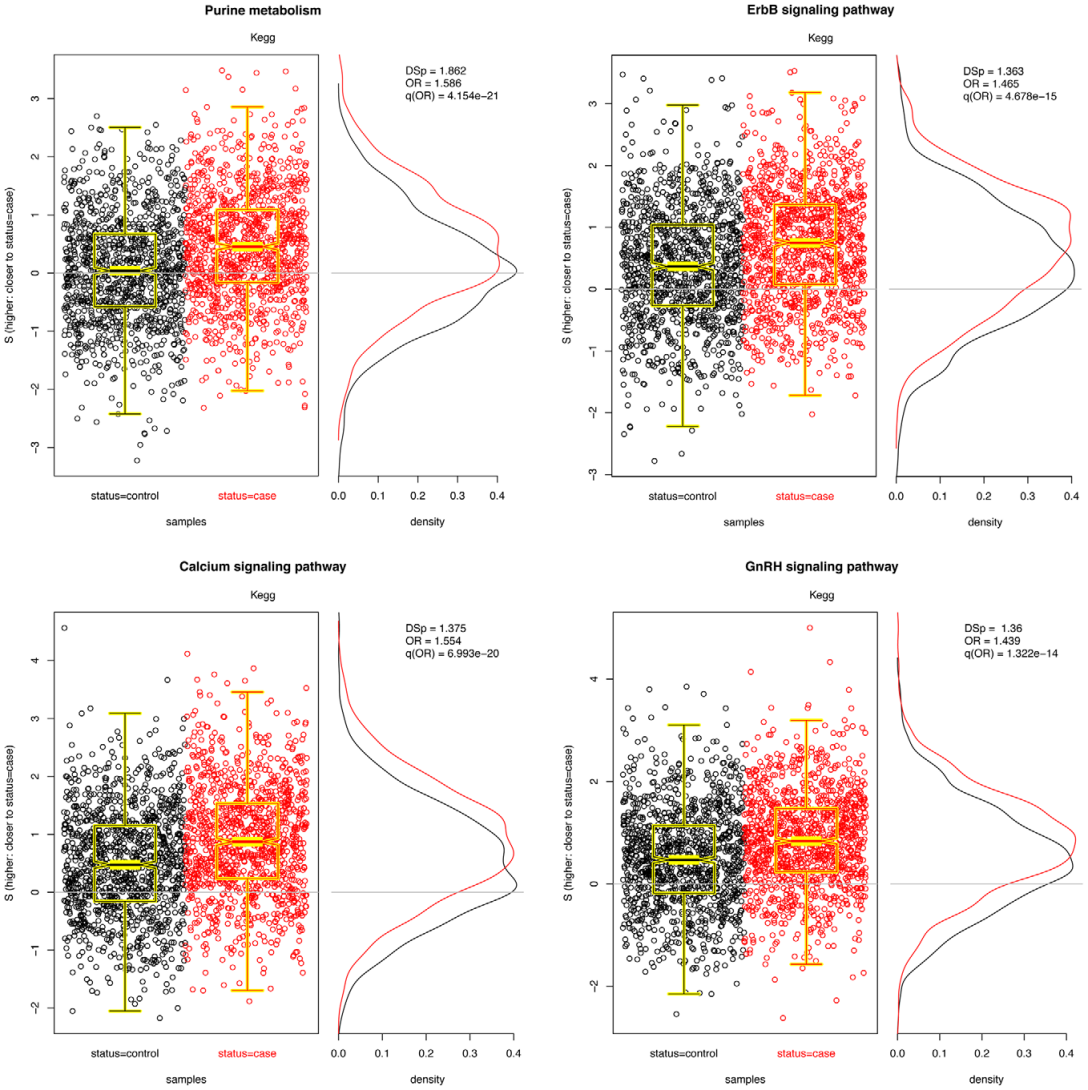
Four significant pathways in breast cancer data. Scatter plots of 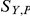 for each pathway are overlayed with boxplots are given in the left panel; higher values of 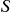 indicate that the sample is closer to other cases than it is to other controls. Distributions of 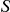 for cases (red) and controls (black) are given to the right. A significant shift toward higher 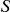 values is seen in the cases. Odds ratios and FDR-adjusted OR 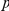 values are given.

Pathways with significant 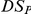 and odds ratios are reported in Table 2 and plots of 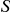 for four of them are illustrated in Figure 3. Although the cases and controls are not crisply separable, a unit increase in 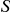 over its range from approximately −3 to 3 yields between a 1.5 and 2.0-fold increase in odds. Importantly, given known minor allele frequencies for cases and controls for this set of SNPs, we can model the increase in odds for an unknown individual based on her “closeness” to other cases.

**Table 2:** PID pathways with significant 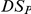 in the CGEMS breast cancer GWAS.

Pathway	Source	Length	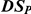	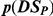	O.R.	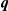 (O.R.)
Purine metabolism	Kegg	136	1.86	6.36e-03	1.59	4.15e-21
Calcium signaling pathway	Kegg	100	1.38	1.82e-03	1.55	6.99e-20
Melanogenesis	Kegg	84	2.36	4.55e-03	1.53	1.47e-18
Gap junction	Kegg	80	1.54	5.45e-03	1.49	1.49e-16
ErbB signaling pathway	Kegg	81	1.36	1.45e-02	1.46	4.68e-15
Long-term potentiation	Kegg	60	1.71	9.09e-04	1.45	4.34e-15
GnRH signaling pathway	Kegg	79	1.36	1.18e-02	1.44	1.32e-14
TCR signaling in naive CD4+ T cells	NCI-Nature	60	2.11	5.45e-03	1.42	7.80e-13
Prostate cancer	Kegg	75	1.45	4.09e-02	1.38	4.37e-11
PKC-catalyzed phosphorylation myosin phosphatase	BioCarta	20	1.97	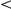 1e-04	1.30	5.82e-09
CCR3 signaling in eosinophils	BioCarta	21	1.59	1.09e-02	1.29	8.86e-08
Biosynthesis of unsaturated fatty acids	Kegg	18	1.69	2.45e-02	1.26	1.38e-06
Attenuation of GPCR signaling	BioCarta	11	1.75	1.09e-02	1.25	2.41e-06
Stathmin and breast cancer resistance to antimicrotubule agents	BioCarta	18	1.84	4.82e-02	1.24	4.96e-06
Visual signal transduction: Cones	NCI-Nature	20	1.56	4.73e-02	1.24	2.24e-06
Dentatorubropallidoluysian atrophy (DRPLA)	Kegg	11	1.84	2.73e-03	1.24	2.24e-06
Intrinsic prothrombin activation pathway	BioCarta	22	1.35	3.18e-02	1.23	4.61e-06
Eicosanoid metabolism	BioCarta	19	1.69	1.91e-02	1.23	3.44e-06
Effects of botulinum toxin	NCI-Nature	7	1.44	2.27e-02	1.20	3.50e-05
Activation of PKC through G-protein coupled receptors	BioCarta	10	1.50	9.09e-03	1.20	8.42e-06
Streptomycin biosynthesis	Kegg	9	1.36	3.55e-02	1.17	1.89e-04
PECAM1 interactions	Reactome	6	2.70	5.45e-03	1.17	7.28e-05
HDL-mediated lipid transport	Reactome	8	1.47	2.00e-02	1.14	1.59e-03
Granzyme A mediated apoptosis pathway	BioCarta	8	1.97	1.73e-02	1.12	6.60e-04

(Pathways with over 60% SNPs covered by another pathway have been removed; for the complete list, see Table S1). Pathway-length based resampled 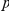-values, denoted 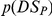, are given for significant pathways, along with the odds ratios and associated FDRs for a logistic regression model.

In order to ensure that the pathways listed were not interrogating the same set of genes, we carried out two checks. First, we computed the SNP overlap between all pairs of significant pathways, sequentially removing pathways that shared in excess of 60% of their genes with another pathway. Because this is done using a greedy algorithm that depends on the order of the pathways input, the culling algorithm was run with different starting orders, and the most frequent output was kept. No pathway remaining in Table 2 shares more than 60% of its SNPs with another pathway. (An un-culled list may be found in Table S1.) Second, we computed the correlation of 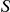 values between each pair of pathways to assess whether any pathway's 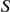 statistic was reflecting the same genetic variation as another (i.e., whether samples that had high 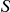 values for one pathway consistently did so in another). The maximum correlation of 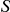 values observed between any two pathways in Table 2 was 0.58, suggesting that a different subset of samples is affected in each pathway.

Many of the pathways listed in Table 2 fulfill biological functions that are well known to be cancer-associated, playing a strong role in cell proliferation and migration, processes which are perturbed in malignancies. Purine metabolism–the most significantly associated pathway–has been observed to be altered in cancer cells [39], [40], and the majority of the other significant pathways are directly related to cell migration (e.g., ErbB signaling and gap junction pathways) and cellular signalling (e.g., calcium signaling, PKC-catalyzed phosphorylation of myosin phosphatase, attenuation of GPCR signaling, and activation of PKC through GPCRs) processes that have been implicated in a variety of cancers. In addition, eicosanoids and unsaturated fatty acid metabolism have been associated with breast cancer specifically [41]. In general, the findings in Table 2 suggest that there exist germline genetic differences in these mechanisms that confer a predisposition to disease.

Interestingly, the GnRH (gonadotropin releasing hormone) signaling pathway appears to be significant. GnRH has been linked with HR-positive breast cancer and the use of GnRH analogues in breast cancer treatment has already been proposed [42], [43]. However, a recent large sequencing study found no association of GnRH1 or GnRH receptor gene polymorphisms with breast cancer risk [44], contrary to the author's hypothesis that common, functional polymorphisms of GnRH1 and GnRHR could influence breast cancer risk by modifying hormone production. In contrast to their null findings, our result suggests that there are system-wide variations in GnRH signalling that contribute to risk that are not evident when considering the GnRH1 and GnRHR SNPs independently.

Of the 1081 pathways considered, four–FGF signaling, MAPK signaling, regulation of actin cytoskeleton, and prostate cancer–contained 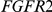, the gene found to be significantly associated in the initial CGEMS analysis [4]. However, only one–prostate cancer–was significant in comparison to randomly generated pathways of the same length. It may reasonably be asked, then, whether the high significance of the prostate cancer pathway in Table 2 is a result of 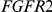. To address this, we eliminated the 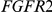 SNP from the prostate cancer pathway; the resampling-based test remained significant (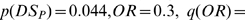 8.2e-09), suggesting that the association of the prostate cancer pathway is not driven solely by differences in 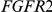.

### Liver cancer GWAS results

**Figure 4:**
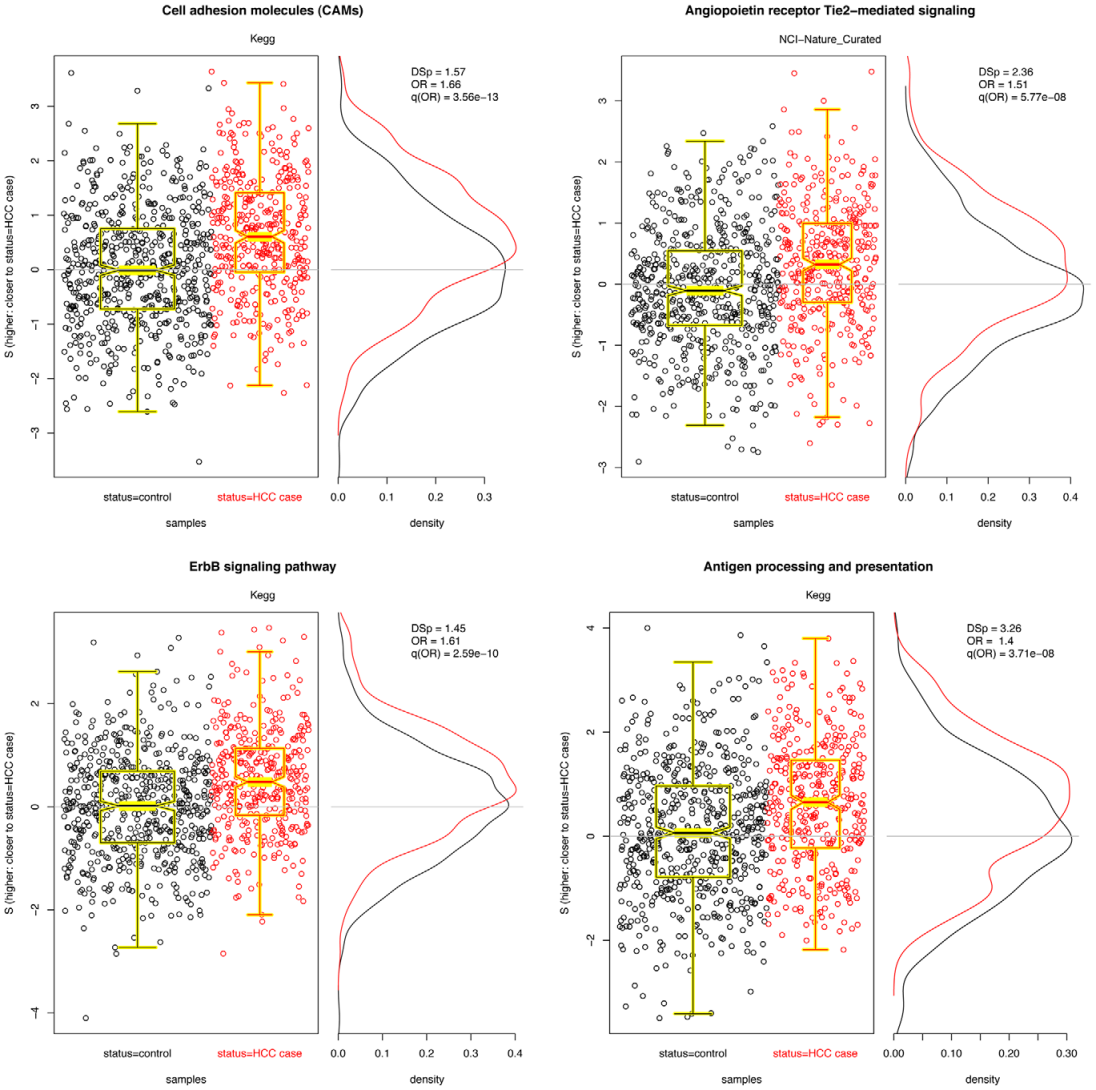
Four significant pathways in liver cancer data. Scatter plots of 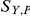 for each pathway are overlayed with boxplots are given in the left panel; higher values of 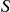 indicate that the sample is closer to other cases than it is to other controls. Distributions of 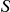 for cases (red) and controls (black) are given to the right. A significant shift toward higher 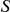 values is seen in the cases. Odds ratios and FDR-adjusted OR 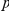 values are given.

We carried out the same procedure in using data from the liver cancer GWAS described above. Here, 1049 pathways were non-trivially covered in the data set; 53079 SNPs in the data could be associated with at least one of the pathways. Because these 53079 SNPs were associated with 3718 unique genes, 3718 were kept in the analysis (the most significant SNP for each gene of interest). The 1081 pathways ranged from 2 to 193 genes, with a mean of 16. As above, 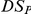 scores for differential 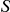 distributions in cases and controls were computed for each pathway, resampled 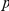 values obtained for each pathway size, odds ratios for 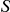 were obtained, and the multiple hypotheses were corrected using FDR adjustment [37], [38]. Significant pathways are listed in Table 3, and plots of the top three pathways are given in Figure 4a–4d. As in the breast cancer data above, we removed pathways which had over 60% their SNPs covered by another pathway (a complete list, with overlapping pathways, is give in Table S2) and examined the correlation in 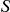 for all remaining pathways (maximum 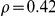).

**Table 3:** PID pathways with significant 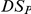 in the liver cancer GWAS.

Pathway	Source	Length	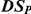	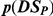	O.R.	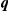 (O.R.)
Cell adhesion molecules (CAMs)	Kegg	86	1.57	9.09e-03	1.66	3.56e-13
ErbB signaling pathway	Kegg	76	1.45	3.45e-02	1.61	2.59e-10
Signaling events mediated by Stem cell factor receptor (c-Kit)	NCI-Nature	40	2.35	5.45e-03	1.58	7.31e-10
Neurotrophic factor-mediated Trk receptor signaling	NCI-Nature	50	1.60	2.36e-02	1.55	2.49e-08
Lissencephaly gene (LIS1) in neuronal migration and development	NCI-Nature	21	2.02	7.27e-03	1.52	1.44e-07
Angiopoietin receptor Tie2-mediated signaling	NCI-Nature	40	2.36	1.36e-02	1.51	5.77e-08
Reelin signaling pathway	NCI-Nature	28	1.62	5.45e-03	1.46	7.35e-08
Syndecan-4-mediated signaling events	NCI-Nature	27	1.74	1.64e-02	1.46	1.19e-06
Galactose metabolism	Kegg	19	1.65	2.27e-02	1.44	5.01e-06
Vibrio cholerae infection	Kegg	35	1.84	2.64e-02	1.43	6.67e-07
Paxillin-independent events mediated by a4b1 and a4b7	NCI-Nature	19	2.14	1.00e-02	1.40	6.67e-07
Antigen processing and presentation	Kegg	34	3.26	1.36e-02	1.40	3.71e-08
Corticosteroids and Cardioprotection	BioCarta	21	1.98	3.55e-02	1.39	1.24e-05
Lissencephaly gene (Lis1) in neuronal migration and development	BioCarta	15	1.60	1.36e-02	1.37	2.52e-05
IL12 signaling mediated by STAT4	NCI-Nature	25	1.93	4.55e-02	1.37	1.58e-05
Biosynthesis of unsaturated fatty acids	Kegg	13	1.76	1.64e-02	1.36	6.44e-05
Growth hormone signaling pathway	BioCarta	18	1.75	3.18e-02	1.36	7.46e-05
Canonical Wnt signaling pathway	NCI-Nature	28	1.92	4.73e-02	1.35	9.36e-06
NO2-dependent IL-12 pathway in NK cells	BioCarta	8	1.82	2.73e-03	1.32	5.83e-05
Signaling events mediated by HDAC Class III	NCI-Nature	19	2.12	3.91e-02	1.32	4.19e-05
Removal of aminoterminal propeptides from 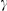 -carboxylated proteins	Reactome	7	3.12	5.45e-03	1.29	8.46e-05
Aminophosphonate metabolism	Kegg	13	1.91	3.36e-02	1.26	8.17e-04
Antigen processing and presentation	BioCarta	6	2.61	1.82e-03	1.22	3.36e-05
Classical complement pathway	BioCarta	12	2.27	1.55e-02	1.19	1.67e-04
Chylomicron-mediated lipid transport	Reactome	7	1.94	3.27e-02	1.16	1.49e-02

(Pathways with over 60% SNPs covered by another pathway have been removed; for the complete list, see Table S2). Pathway-length based resampled 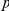-values, denoted 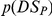, are given for significant pathways, along with the odds ratios and associated FDRs for a logistic regression model.

The results here are interesting. First, we observe that a couple pathways are significant in both the CGEMS breast and liver GWAS with similar effect sizes, namely ErbB signaling and biosynthesis of unsaturated fatty acids. ErbB has a well–established association with cancer; unsaturated fatty acid biosynthesis may link diet to cancer risk, and its appearance may suggest a gene-environment interaction. The commonality of these known cancer-associated pathways across the two studies suggest that there may exist genetic patterns that confer carcinogenesis risk irrespective of the disease site. Along with those shared in the breast cancer data, many of the other significant pathways in the liver cancer data well known to be tumorassociated, including cell adhesion molecules, Wnt signaling, c-Kit receptor, and angiogenesis pathways, further supporting the notion that germline genetic differences in these mechanisms contribute to cancer risk. The appearance of many neuronal pathways is also supported by our understanding of carcinogenesis: thes contain well-known signal transduction molecules including Ras and PKA that may both be driving their conferring increased cancer risk and driving the significance of the pathway [45].

Additionally, six of the 25 significant liver cancer pathways are immune– and inflammation–related, namely, antigen processing and presentation (two, with 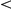
60% overlap), classical complement pathway, corticosteroids, IL12 signaling mediated by STAT4, and NO2-dependent IL-12 pathway in NK cells. This is a particularly interesting finding in light of the fact that the original analysis of the liver data [36] suggested that altered T-cell activation plays a direct role in the onset of liver cancer. The involvement of the immune system in liver cancer development has been established in clinical studies and research involving model organisms. Increased activity of helper T-cells, which promote inflammation, is associated with hepatocellular carcinogenesis [46] while activation and proliferation of cytotoxic T-lymphocytes is suppressed in liver cancers [47], [48]. The inflammatory immune response, mediated by interleukins, has also been closely connected to liver cancers in mice [49] and humans [50]–[52]. These findings, coupled with the observation of several significant immune-related pathways in our data, are suggestive of germline polymorphisms in immune response that lead to hepatocellular carcinoma risk.

### Combining pathways

**Figure 5:**
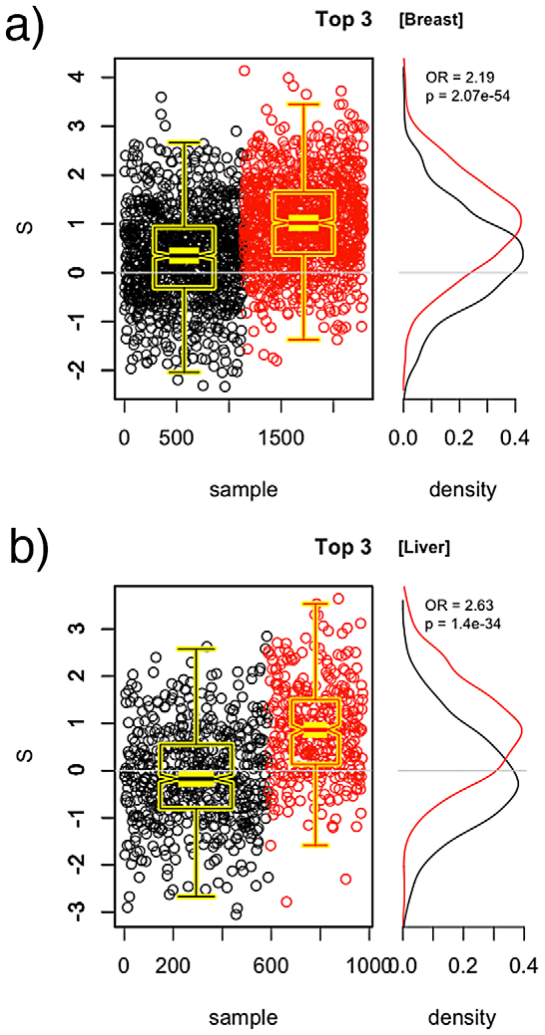
Union of top three pathways. SNPs from the top three pathways are combined to compute 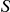 for the breast cancer data (a) and the liver cancer data (b). Distributions of 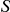 for cases (red) and controls (black) are given to the right. A significant shift toward higher 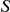 values is seen in the cases.

In both the breast and liver cancer results, we see observe that even though significant pathways yield between a 1.5 and 2.0-fold increase in odds for each unit increase in 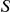 (over its typical range of approximately –3 to 3), the cases and controls are not crisply separable based on 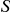 values. These findings suggest that it may be possible to combine pathways to yield a model that is more predictive than a single pathway alone. However, the 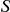 values must not simply be put into the regression model because the overlap in pathways will result in some SNPs being double-counted. Rather, we combine pathways by taking the union of their SNPs, and recomputing the statistics. Doing this sequentially for the top pathways in the order as listed in Table 2 and Table 3 yields the values given in Table 4 and Table 5, respectively. Considerably higher ORs are obtained when combining the significant pathways. An illustration of the case and control distributions when using a “superpathway” comprised of the top three pathways in the breast and liver data, respectively, is given in Figure 5. These findings support the notion that the genomic variation contributing to risk is spread over several mechanisms, rather than being concentrated in a single gene.

**Table 4:** PoDA results for sucessive unions of significant pathways in the CGEMS breast cancer data.

Pathway	Length	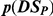	O.R.	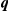 (O.R.)
Top-2	318	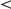 1e-04	2.02	1.63e-46
Top-3	397	1.00e-04	2.19	2.07e-54
Top-4	474	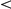 1e-04	2.33	3.65e-62
Top-5	522	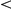 1e-04	2.45	6.83e-66
Top-6	544	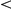 1e-04	2.44	8.51e-66
Top-7	558	2.00e-04	2.47	1.22e-67
Top-8	626	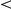 1e-04	2.59	1.01e-73
Top-9	658	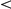 1e-04	2.64	9.84e-75
Top-10	700	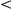 1e-04	2.77	9.72e-79
Top-11	710	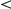 1e-04	2.80	1.42e-79
Top-12	723	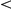 1e-04	2.82	2.06e-80
Top-13	739	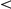 1e-04	2.89	3.31e-82
Top-14	744	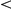 1e-04	2.93	2.86e-83
Top-15	770	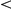 1e-04	2.96	6.41e-85
Top-16	774	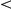 1e-04	2.97	5.10e-85
Top-17	791	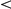 1e-04	2.95	2.43e-85
Top-18	800	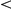 1e-04	3.06	1.15e-87
Top-19	814	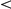 1e-04	3.14	1.19e-89
Top-20	832	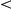 1e-04	3.26	4.51e-92
Top-21	837	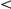 1e-04	3.28	2.92e-92
Top-22	839	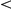 1e-04	3.29	2.41e-92
Top-23	845	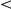 1e-04	3.34	1.45e-93
Top-24	854	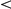 1e-04	3.38	4.62e-95

Pathway-length based resampled 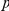 values, denoted 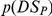, are given along with the odds ratios and associated FDRs for a logistic regression model.

**Table 5:** PoDA results for sucessive unions of significant pathways in the liver cancer data.

Pathway	Length	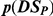	O.R.	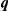 (O.R.)
Top-2	321	5.38e-02	2.37	1.20e-27
Top-3	402	2.80e-03	2.63	1.40e-34
Top-4	474	1.10e-03	2.86	6.50e-38
Top-5	539	9.00e-04	3.22	4.03e-42
Top-6	560	1.00e-04	3.39	1.19e-43
Top-7	580	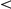 1e-04	3.50	1.39e-44
Top-8	589	6.00e-04	3.50	1.35e-44
Top-9	603	4.00e-04	3.52	1.23e-44
Top-10	624	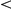 1e-04	3.60	1.33e-45
Top-11	640	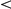 1e-04	3.73	3.69e-47
Top-12	646	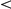 1e-04	3.78	1.68e-47
Top-13	667	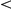 1e-04	3.81	9.29e-48
Top-14	709	3.00e-04	3.88	1.90e-48
Top-15	751	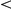 1e-04	4.09	2.11e-49
Top-16	761	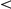 1e-04	4.09	1.76e-49
Top-17	797	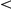 1e-04	4.45	1.29e-50
Top-18	805	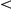 1e-04	4.46	5.24e-51
Top-19	823	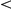 1e-04	4.56	2.20e-51
Top-20	838	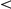 1e-04	4.56	1.73e-51

Pathway-length based resampled 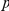 values, denoted 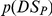, are given along with the odds ratios and associated FDRs for a logistic regression model.

## Discussion

We have introduced the Pathways of Distinction analysis method (PoDA) for identifying pathways which can be used to distinguish between phenotype groups. PoDA identifies sets of SNPs in GWAS studies for which cases and controls exhibit differential “closeness” to other cases and controls; that is, it permits one to infer whether cases are more similar to other cases than are controls across a given set of SNPs. Because PoDA is designed to detect the joint effects of multiple SNPs, it presents an approach to GWAS analysis that augments single-SNP or single-gene tests.

We applied PoDA to two GWAS data sets, with highly promising results. In the breast cancer data, we found a number of pathways which are known to play a role in cancers generally and breast cancer specifically, suggesting that differences in these mechanisms which confer disease risk may exist at the germline DNA level. In the liver cancer data, we found an extreme abundance of immune-related pathways, further corroborating the known link between inflammation and hepatocellular carcinoma, and bolstering the observation in [36] that germ-line differences in immune function may play a role in liver carcinogenesis.

PoDA may be used as a complement to other multi-SNP analysis techniques [18]–[21]. Unlike gene-set enrichment type approaches [17]–[19], which search for an overabundance of significant markers in a gene set of interest, PoDA finds both sets containing highly significant markers as well as sets that have a subtle but consistent pattern across all the markers in the set. This permits the detection of pathways in which the joint action of several alterations produce a phenotype and those for which any of several possible alterations, none of them the dominant one, confer predisposition to disease. Indeed, many of the pathways indicated in our analysis of the breast cancer data (Table 2) were not detected using SNP-set enrichment [17]–[19] (data not shown), including the highly significant purine metabolism and GnRH signaling pathways, both of which are biologically relevant (purine metabolism has been implicated in cancers generally due to its role in DNA and RNA synthesis [40], and GnRH has been shown to be clinically important in breast and gynecological cancers [53]). These pathways, along with others that were indicated using PoDA but not enrichment analysis (data not shown), have a statistically significant difference in case and control 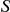 distributions and remain significant in comparison with randomly-generated pathways of the same length.

Because PoDA effectively measures the closeness of each individual to remaining cases and controls, it bears a conceptual relationship to nearest-neighbor and nearest-centroid classifiers [54], [55], as well as to the distance-based feature selection algorithms like Relief-F and its derivatives [25]–[28]. However, it must be remembered that the goal of PoDA is to indicate *mechanisms* that may be deleteriously hit at the genomic level even when those hits are heterogeneous, whereas the goal of nearest-centroid classifiers and Relief-F–type feature selection is to derive a minimal set of markers that best classify cases and controls (and thus are the most homogeneously hit). These approaches are complementary, and one can easily envision an application in which (e.g.) Relief-F is run *within* pathways that are highly significant in the PoDA analysis in order to single out the SNPs driving the effect. In fact, this approach may improve ReliefF's ability to find those genes, since the nearest neighbors from which the Relief SNP weights are calculated would be the nearest-neighbors for that specific pathway, thus discounting heterogeneity introduced by mechanistically unrelated genes. For instance, in the provided example (Figure 1), ReliefF fails to identify the significance of SNPs 1–3 when run using the complete 50-SNP data, but places at least two of SNPs 1, 2 or 3 in the top third of selected features when restricted to SNPs 1–12.

While PoDA has many benefits, it should be noted that when epistasis drives a phenotype with *no* differences in the minor allele frequencies for the epistatically-interacting genes (as opposed to a slight yet consistent one shown in the example), PoDA as computed via Eqs. 1,2 will miss the pathway. Geometrically, such a situation would mean that the case and control groups have the same centroids while having a different distribution of samples about those centroids. A famous example of this is provided through the non-linearly separable XOR (exclusive or): consider two epistatic loci 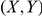 such that all controls have genotypes in the set 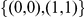 and all cases have genotypes in the set 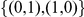 (i.e., that a genotype of 1 at either locus can be compensated by a genotype of 1 at the other, but having just one alone–1 at exclusively 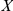 or 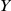
–is deleterious). If the loci 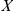 and 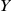 each have the same MAF in cases and controls, it is plain to see that the centroids will be in the same location for both groups, and Eq. 1 will yield zero for both cases and controls. If instead of using Eq. 1, we compute pairwise sample-sample distances, we can circumvent this limitation and find such epistatic situations (it is this pairwise approach that permits Relief-F to also uncover nonlinearly interacting SNPs). While we provide the facility for this in the PoDA package, the cost of carrying out the pairwise computation is a considerable increase in computational complexity.

A number of potential avenues exist to extend the application of PoDA further. One possible application is in improving the reproducibility of GWAS results. We note that several of the pathways identified in the breast cancer GWAS data were also implicated in the liver cancer data, which suggests that there may be common features which distinguish individuals to cancer generally. Because different GWA studies–even those of the same phenotypes–often yield different results at the SNP level, it may be possible to find common alterations at the pathway level across disparate GWAS using PoDA.

Extending PoDA further, the 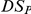 scores obtained for each pathway may be examined for over-representation of extreme values in pathways that comprise a particular biological subsystem–one may think of this as a “pathway-set” enrichment analysis (which would be conducted using the a running-sum statistic analogous GSEA [17]), and could use it to answer whether (e.g.) immune-related pathways are hit in liver cancer more often than expected by chance. Alternatively, boosting [56], [57] could be used to find sets of pathways which are more predictive of case status than individual pathways. Either of these approaches would yield a richer, systems-wide view of the connection between genotype and phenotype. Finally, because PID contains topological information regarding pathway connectivity, one may consider sub-networks of pathways, permitting one to find potential chemopreventive and therapeutic targets. Alternatively, Relief-F can be used, as mentioned above, in a pathway–specific manner to yield the subset of SNPs that drive the distinction of cases and controls in high-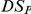 pathways.

PoDA provides an advantage over existing GWAS analysis methods. Because it does not rely on the significance of individual markers, it has the power to aid in identifying the genomic causes of complex diseases that would not be detected in single-gene tests or enrichment analyses. The size of the SNP set is not limited in PoDA, and since PoDA leverages known biological relationships to find multi-SNP effects, the results are readily interpretable. PoDA may thus be used to augment existing analysis techniques and provide a richer, systems-level understanding of genomics.

### Availability

R software to carry out the PoDA computation is available via http://braun.tx0.org/PoDA.

## Supporting Information

Table S1Full list PID pathways with significant 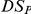 in the breast cancer GWAS, including highly “overlapping” pathways. Pathway-length based resampled 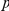-values, denoted 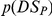, are given for significant pathways, along with the odds ratios and associated FDRs for a logistic regression model.(PDF)

Table S2Full list PID pathways with significant 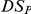 in the liver cancer GWAS, including highly “overlapping” pathways. Pathway-length based resampled 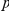-values, denoted 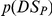, are given for significant pathways, along with the odds ratios and associated FDRs for a logistic regression model.(PDF)
