# Supplementary material for: Pathways of Distinction Analysis: A New Technique for Multi–SNP Analysis of GWAS Data
Source: PLoS Genet. 2011 Jun 9;7(6):e1002101. doi: 10.1371/journal.pgen.1002101 (PMC3111473; doi:10.1371/journal.pgen.1002101)
Supplement: Table S1 — Full list PID pathways with significant in the breast cancer GWAS, including highly “overlapping” pathways. Pathway-length based resampled -values, denoted , are given for significant pathways, along with the odds ratios and associated FDRs for a logistic regression model. (PDF) [file pgen.1002101.s001.pdf]

Pathway	Source	Length	DS_P	$p(DS_P)$	O.R.	q(O.R.)
Purine metabolism	Kegg	136	1.86	6.36e-03	1.59	4.15e-21
Calcium signaling pathway	Kegg	100	1.38	1.82e-03	1.55	6.99e-20
Melanogenesis	Kegg	84	2.36	4.55e-03	1.53	1.47e-18
Gap junction	Kegg	80	1.54	5.45e-03	1.49	1.49e-16
ErbB signaling pathway	Kegg	81	1.36	1.45e-02	1.46	4.68e-15
Long-term potentiation	Kegg	60	1.71	9.09e-04	1.45	4.34e-15
GnRH signaling pathway	Kegg	79	1.36	1.18e-02	1.44	1.32e-14
TCR signaling in naive CD4+ T cells	NCI-Nature	60	2.11	5.45e-03	1.42	7.80e-13
TCR signaling in naive CD8+ T cells	NCI-Nature	48	2.03	7.27e-03	1.38	1.11e-11
Prostate cancer	Kegg	75	1.45	4.09e-02	1.38	4.37e-11
PKC-catalyzed phosphorylation ... myosin phosphatase	BioCarta	20	1.97	<1e-04	1.30	5.82e-09
CCR3 signaling in eosinophils	BioCarta	21	1.59	1.09e-02	1.29	8.86e-08
Biosynthesis of unsaturated fatty acids	Kegg	18	1.69	2.45e-02	1.26	1.38e-06
Attenuation of GPCR signaling	BioCarta	11	1.75	1.09e-02	1.25	2.41e-06
Stathmin and breast cancer resistance to antimicrotubule agents	BioCarta	18	1.84	4.82e-02	1.24	4.96e-06
Visual signal transduction: Cones	NCI-Nature	20	1.56	4.73e-02	1.24	2.24e-06
Dentatorubropallidoluysian atrophy (DRPLA)	Kegg	11	1.84	2.73e-03	1.24	2.24e-06
Intrinsic prothrombin activation pathway	BioCarta	22	1.35	3.18e-02	1.23	4.61e-06
Eicosanoid metabolism	BioCarta	19	1.69	1.91e-02	1.23	3.44e-06
Effects of botulinum toxin	NCI-Nature	7	1.44	2.27e-02	1.20	3.50e-05
Activation of PKC through G-protein coupled receptors	BioCarta	10	1.50	9.09e-03	1.20	8.42e-06
Ca-calmodulin-dependent protein kinase activation	BioCarta	8	1.70	1.00e-02	1.19	5.67e-05
Streptomycin biosynthesis	Kegg	9	1.36	3.55e-02	1.17	1.89e-04
PECAM1 interactions	Reactome	6	2.70	5.45e-03	1.17	7.28e-05
HDL-mediated lipid transport	Reactome	8	1.47	2.00e-02	1.14	1.59e-03
Granzyme A mediated apoptosis pathway	BioCarta	8	1.97	1.73e-02	1.12	6.60e-04

Supplementary Table S-1.
