# Supplementary material for: Pathways of Distinction Analysis: A New Technique for Multi–SNP Analysis of GWAS Data
Source: PLoS Genet. 2011 Jun 9;7(6):e1002101. doi: 10.1371/journal.pgen.1002101 (PMC3111473; doi:10.1371/journal.pgen.1002101)
Supplement: Table S2 — Full list PID pathways with significant in the liver cancer GWAS, including highly “overlapping” pathways. Pathway-length based resampled -values, denoted , are given for significant pathways, along with the odds ratios and associated FDRs for a logistic regression model. (PDF) [file pgen.1002101.s002.pdf]

Pathway	Source	Length	DS_P	$p(DS_P)$	O.R.	q(O.R.)
Cell adhesion molecules (CAMs)	Kegg	86	1.57	9.09e-03	1.66	3.56e-13
ErbB signaling pathway	Kegg	76	1.45	3.45e-02	1.61	2.59e-10
Signaling events mediated by Stem cell factor receptor (c-Kit)	NCI-Nature	40	2.35	5.45e-03	1.58	7.31e-10
Neurotrophic factor-mediated Trk receptor signaling	NCI-Nature	50	1.60	2.36e-02	1.55	2.49e-08
Lissencephaly gene (LIS1) in neuronal migration and development	NCI-Nature	21	2.02	7.27e-03	1.52	1.44e-07
Angiopoietin receptor Tie2-mediated signaling	NCI-Nature	40	2.36	1.36e-02	1.51	5.77e-08
Reelin signaling pathway	NCI-Nature	28	1.62	5.45e-03	1.46	7.35e-08
Syndecan-4-mediated signaling events	NCI-Nature	27	1.74	1.64e-02	1.46	1.19e-06
Galactose metabolism	Kegg	19	1.65	2.27e-02	1.44	5.01e-06
TPO signaling pathway	BioCarta	17	2.61	6.36e-03	1.44	3.80e-06
Vibrio cholerae infection	Kegg	35	1.84	2.64e-02	1.43	6.67e-07
Paxillin-independent events mediated by a4b1 and a4b7	NCI-Nature	19	2.14	1.00e-02	1.40	6.67e-07
Antigen processing and presentation	Kegg	34	3.26	1.36e-02	1.40	3.71e-08
Corticosteroids and cardioprotection	BioCarta	21	1.98	3.55e-02	1.39	1.24e-05
Lissencephaly gene (Lis1) in neuronal migration and development	BioCarta	15	1.60	1.36e-02	1.37	2.52e-05
IL12 signaling mediated by STAT4	NCI-Nature	25	1.93	4.55e-02	1.37	1.58e-05
Biosynthesis of unsaturated fatty acids	Kegg	13	1.76	1.64e-02	1.36	6.44e-05
Growth hormone signaling pathway	BioCarta	18	1.75	3.18e-02	1.36	7.46e-05
Canonical Wnt signaling pathway	NCI-Nature	28	1.92	4.73e-02	1.35	9.36e-06
NO2-dependent IL-12 pathway in nk cells	BioCarta	8	1.82	2.73e-03	1.32	5.83e-05
Signaling events mediated by HDAC Class III	NCI-Nature	19	2.12	3.91e-02	1.32	4.19e-05
Removal of aminoterminal propeptides from gamma-carboxylated proteins	Reactome	7	3.12	5.45e-03	1.29	8.46e-05
Gamma-carboxylation, transport, and amino-terminal cleavage of proteins	Reactome	6	3.25	1.82e-03	1.28	6.64e-05
Transport of γ-carboxylated protein precursors ...	Reactome	6	3.25	1.82e-03	1.28	6.64e-05
Paxillin-dependent events mediated by a4b1	NCI-Nature	17	1.84	2.00e-02	1.28	3.41e-05
Gamma-carboxylation of protein precursors	Reactome	7	2.86	3.64e-03	1.28	1.38e-04
Aminophosphonate metabolism	Kegg	13	1.91	3.36e-02	1.26	8.17e-04
Antigen processing and presentation	BioCarta	6	2.61	1.82e-03	1.22	3.36e-05
Lectin induced complement pathway	BioCarta	11	1.91	2.18e-02	1.20	1.55e-04
Classical complement pathway	BioCarta	12	2.27	1.55e-02	1.19	1.67e-04
Chylomicron-mediated lipid transport	Reactome	7	1.94	3.27e-02	1.16	1.49e-02

Supplementary Table S-2.
